# The Putative Histone Methyltransferase DOT1 Regulates Aflatoxin and Pathogenicity Attributes in *Aspergillus flavus*

**DOI:** 10.3390/toxins9070232

**Published:** 2017-07-24

**Authors:** Linlin Liang, Yinghang Liu, Kunlong Yang, Guinan Lin, Zhangling Xu, Huahui Lan, Xiuna Wang, Shihua Wang

**Affiliations:** Fujian Key Laboratory of Pathogenic Fungi and Mycotoxins, Key Laboratory of Biopesticide and Chemical Biology of Education Ministry, and School of Life Sciences, Fujian Agriculture and Forestry University, Fuzhou 350002, China; lllyiyuan@sina.com (L.L.); lyinghang35324@163.com (Y.L.); ykl_long@yeah.net (K.Y.); linguinan1996@outlook.com (G.L.); xuzhangling0409@163.com (Z.X.); huahuilan90@sina.com (H.L.); Wangxn@cau.edu.cn (X.W.)

**Keywords:** histone methylation, sclerotia, stress response, fungal virulence

## Abstract

Lysine methyltransferases transfer methyl groups in specific lysine sites, which regulates a variety of important biological processes in eukaryotes. In this study, we characterized a novel homolog of the yeast methyltransferase DOT1 in *A*. *flavus*, and observed the roles of *dot1* in *A*. *flavus*. Deletion of *dot1* showed a significant decrease in conidiation, but an increase in sclerotia formation. A change in viability to multiple stresses was also found in the Δ*dot1* mutant. Additionally, aflatoxin (AF) production was found severely impaired in the Δ*dot1* mutant. Further analysis by qRT-PCR revealed that the transcription of AF structural genes and their regulator gene *aflS* were prominently suppressed in the Δ*dot1* mutant. Furthermore, our data revealed that Dot1 is important for colonizing maize seeds in *A*. *flavus*. Our research indicates that Dot1 is involved in fungal development, aflatoxin biosynthesis and fungal virulence in *A*. *flavus*, which might provide a potential target for controlling *A*. *flavus* with new strategies.

## 1. Introduction

The ubiquitous fungus *A. flavus* is notorious for its contamination of many foodstuffs and important crops, such as maize, peanuts and rice, during pre- or post-harvest with the most carcinogenic mycotoxin, aflatoxin [1,2,3]. This fungus is also known as the second most frequent *Aspergillus* pathogen after *A*. *fumigatus* on immunosuppressed patients, which is also responsible for aspergillosis diseases or liver cancer for human and animals caused by the consumption of contaminated food [1,2,4]. Due to this, this fungus has caused enormous agricultural economic losses, food shortages and health problems all cross the world [1,5]. Thus, effective measures to restrict this pathogen are urgently needed, not only to protect human and animal health, but also to alleviate its deleterious effects on agricultural economic losses.

It is quite important to develop rational control strategies for *A*. *flavus* underlying the fungal virulence and mycotoxins attributes. Although numerous studies reported that pathogenesis in *A*. *flavus* was connected with fungal development, secondary metabolism and the adaptability to environmental factors [1,6,7,8], much remains to be learned about the fungal pathogenesis. In the past decade, histone posttranslational modifications (HPTMs), such as phosphorylation, ubiquitination, SUMOylation, acetylation and methylation, have been well documented as providing an important link between many biological processes, fungal secondary metabolite biosynthesis and chromatin-based regulation in eukaryotic cells [5,9,10,11]. Among these, histone methylation was one of most thoroughly studied epigenetic process in HPTMs. In particular, it has been shown that histone lysine methylation catalyzed by lysine methyltransferases in histone specific lysine sites, such as H3K4me2/3, is associated with active regions of chromatin, and H3K9me2/3 and H3K27me2/3 are related to repressive regions [12,13,14,15]. In *Neurospora crassa,* DIM-5, responsible for H3K9 methylation, has a close relationship with gene silencing by mediating DNA methylation on particular cytosine sites [16,17]. The H3K36 methyltransferase SET-2 in *N*. *crassa* has also been reported to regulate fungal development [16,18]. H3K4me and H3K27me are addressed as important for fungal development and many secondary metabolites biosynthesize in fungi [9,12]. 

Studies have demonstrated that many gene clusters of secondary metabolites, including the AF gene cluster, are located near telomeres in *Aspergillus flavus* [19,20]. Genes near telomeres, associated with specific chromatin structures, have position effects, resulting in gene silencing [19,21]. HPTMs such as acetylation, methylation, and ubiquitination have been linked to transcriptional silencing. The inactivation of H3K79 methylation or H3K4 methylation is quite important for heterochromatin formation and for maintaining silencing regions of chromatin in budding yeast [21,22,23]. An abnormal expression of the H3K79 methyltransferase gene *dot1* in yeast and mammalian cells leads to a decrease of gene silence [23,24]. In *Aspergillus*, although many studies have been shown that HPTMs regulate fungal development and secondary metabolite biosynthesis [9,10,25,26,27], there has been little progress in addressing the function of histone lysine methylation in *A*. *flavus*. Here, in order to know if H3K79 is involved in AF biosynthesis, we identified a putative H3K79 methyltransferase Dot1 in *A. flavus*, and then observed the potential roles of the *dot1* gene in *A*. *flavus* by addressing the effects of the deletion mutant on the development, conidiation, sclerotia formation, aflatoxin biosynthesis, and pathogenicity of seeds.

## 2. Results

### 2.1. Characterization of Histone H3K79 Methyltransferase in A. flavus

**Figure 1:**
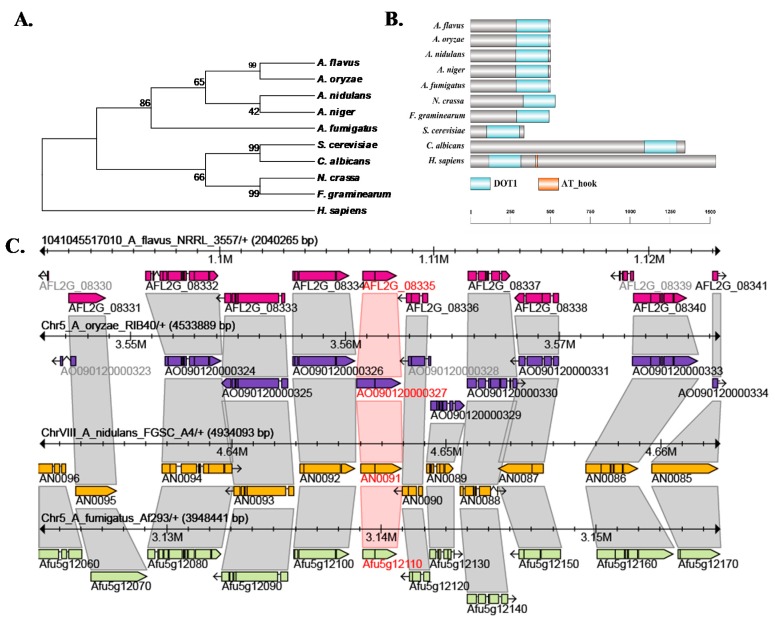
Characterization of H3K79 methyltransferase in *A. flavus*. (**A**) Phylogenetic relationship of Dot1 from different species was analyzed; (**B**) Domains from Dot1 proteins were characterized by SMART, and software DOG 2.0 were used to visualize protein domains; (**C**) The locations and paralogs of *dot1* in *Aspergillus* genomes were analyzed by AspGD (http://www.aspgd.org/).

To characterize the homolog of the *S. cerevisiae* H3K79 methyltransferase Dot1 in *A. flavus,* we used BlastP analyses in the NCBI (https://blast.ncbi.nlm.nih.gov/Blast.cgi?PAGE=Proteins) with the amino acid sequence of *S. cerevisiae* Dot1 (EGA79271.1) as queries. AFLA_093140, encoding a 503 aa protein, was identified in *A. flavus*, which shared a 33% overall identity with *S. cerevisiae* Dot1. The Dot1 protein sequences from different fungi were downloaded, and analyzed their evolutionary relationship by MEGA5.0, which showed that Dot1 from *Aspergillus* Dot1 was highly conserved (Figure 1A). *Dot1* from *Aspergillus* has two exons, and the locations and paralogs of *dot1* were also quite conserved in the *Aspergillus* genomes (Figure 1C). The alignment of Dot1 protein sequences from different fungal species indicates that H3K79 methyltransferase only possesses the DOT1 domain (Figure 1B), however, in *Homo sapiens* the H3K79 methyltransferase has another domain AT_hook, which is a DNA-binding motif with a preference for A/T rich regions (Figure 1B).

### 2.2. Construction of the Deleted (Δdot1) and Complemented (dot1^C^) Mutant Strains

**Figure 2:**
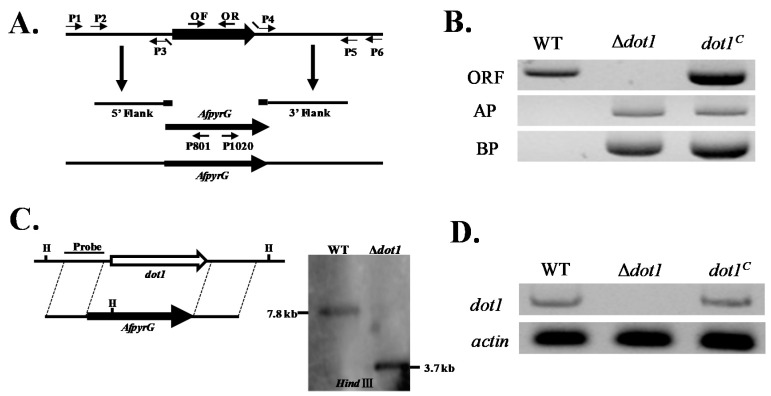
Deletion strategy and confirmation of the mutant strains. (**A**) Deletion strategy for Δ*dot1* using homologous recombination; (**B**) Deleted Δ*dot1* and complemented *dot1^C^* strains were tested by PCR analysis with genomic DNA as template; (**C**) Southern blot analyses of *dot1* deletion mutants using PCR fragment of 5′ flanking region as probes. Genomic DNAs were extracted from WT and putative transformants. Restriction was carried out using the indicated enzymes; (**D**) Δ*dot1* and *dot1^C^* mutant strains were tested by RT-PCR with cDNA as template.

The Δ*dot1* mutants were generated using a homologous recombination strategy as shown in Figure 2A. The selected transformants of Δ*dot1* were analyzed by diagnostic PCR (Figure 2B), and the results showed that a 0.5 kb ORF fragment was amplified from WT, but none from Δ*dot1* (Figure 2B). With gDNA as a template, both 2.0 kb AP and 2.1 kb BP fragments were amplified in Δ*dot1* mutant, but not in wild type (WT) (Figure 2B). The Δ*dot1* mutant then was further confirmed by southern blot analyses (Figure 2C). To confirm that the phenotype changes observed in Δ*dot1* were due to the disruption of *dot1*, the *Δdot1* mutant was complemented with a full-length of *dot1* by a pPTRI plasmid and fused into the Δ*dot1* protoplast. The *dot1^C^* complemented strains were also confirmed by diagnostic PCR using the genomic DNA (Figure 2B) and further verified by RT-PCR, and the result showed that transcription level of Δ*dot1* was not detected in Δ*dot1*, in contrast to the wild type (WT) and *dot1^C^* strains (Figure 2D)*.* All these results indicated that the deletion Δ*dot1* and complemented *dot1^C^* mutant strains were successfully constructed.

### 2.3. Dot1 Is Involved in Fungal Growth and Sporulation

**Figure 3:**
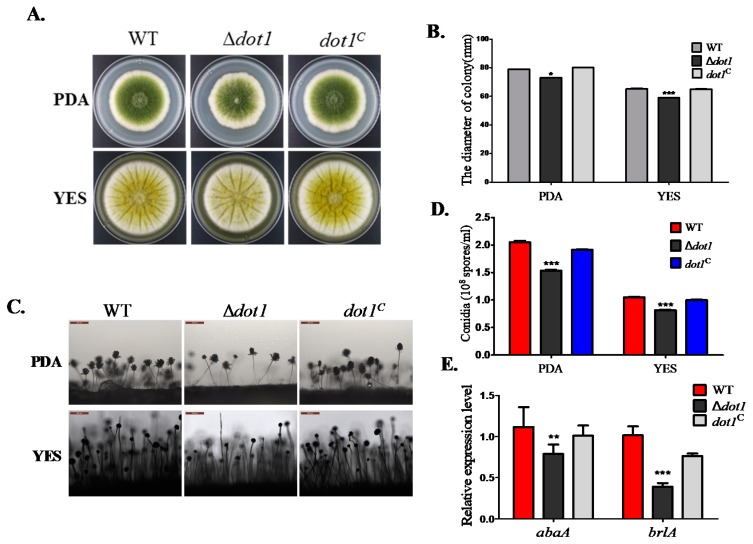
The Δ*dot1* mutant was altered in vegetative growth and conidiation. (**A**) Phenotype of the WT, Δ*dot1* and *dot1^C^* strains after grown on PDA and YES agar plates at 37 °C for 4 days; (**B**) colony diameter in on PDA and YES cultures of the WT strain and mutants were assayed; (**C**) Conidiophores was detected by microscope after light induction; (**D**) The amount of conidia from different strains were determined; (**E**) Transcriptional levels of conidia transcriptional factors genes in the Δ*dot1* mutant, WT and *dot1^C^* strains. *, ** and *** represent significantly different (*p* ≤ 0.05, *p* ≤ 0.01 or *p* ≤ 0.001, respectively). The experiments were conducted with technical triplicates for each strain, and were repeated three times.

To know the potential role of the H3K79 methyltransferase gene *dot1* in fungal development in *A. flavus*, the Δ*dot1,* WT and *dot1^C^* strains were inoculated on YES and PDA in the dark at 37 °C, and the results showed that the Δ*dot1* mutant displayed less pigmentation and marginally decreased in radial growth compared to *dot1^C^* or WT strains (Figure 3A,B). The Δ*dot1* mutant also displayed a significant decrease in conidiation compared to the WT or *dot1^C^* strain (*p* < 0.01) (Figure 3D). Further examination in sporogenesis also showed that the Δ*dot1* mutant produced less normal conidiophore compared to the WT and *dot1^C^* strain (Figure 3C). qRT-PCR was performed to detect the transcriptional expression levels of the conidia transcriptional factors genes *brlA* and *abaA*, which showed that the transcriptional levels of these two conidia specifie genes were both down-regulated in the Δ*dot1* mutant (Figure 3E). These results demonstrate that Dot1 contributes to radical growth and conidiation in *A. flavus*.

### 2.4. Dot1 Plays a Negative Role in Sclerotial Reproduction in A. flavus

**Figure 4:**
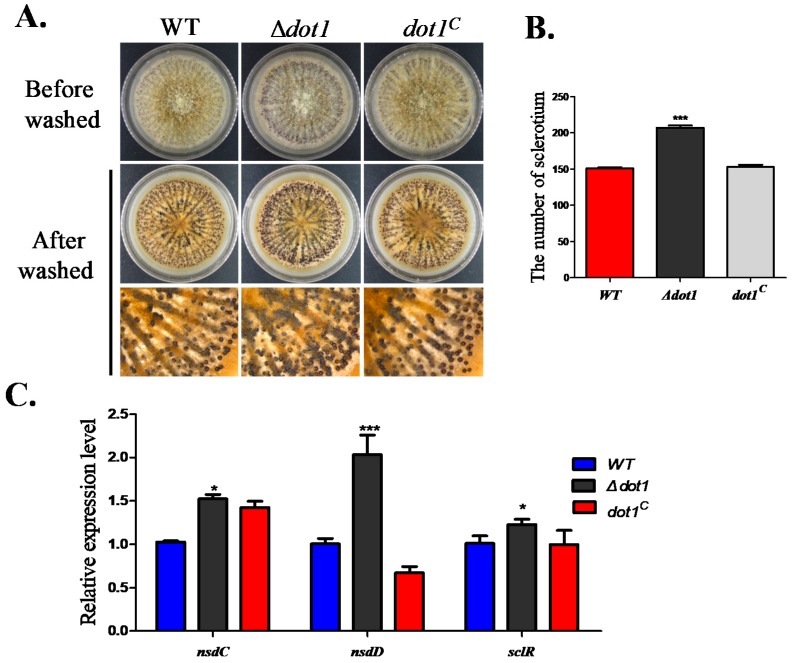
Sclerotia reproduction analysis among the WT, Δ*dot1* and *dot1*^C^ strains. (**A**) Phenotype of the WT, Δ*dot1* and *dot1*^C^ strains were determined after being grown on sclerotia-inducing media, Wickerham medium. The plates were sprayed with 70% ethanol to allow visualization of sclerotia; (**B**) The amount of sclerotia was measured in (**A**); (**C**) Transcriptional levels of the sclerotial specific gene *nsdC*, *nsdD* and *sclR*. * and *** represent *p* ≤ 0.05 and *p* ≤ 0.001, respectively. The experiments were performed with four biological replicates for each strain, and were repeated three times.

Sclerotia is considered to be a vestige of the cleistothecia, which is a survival structure for *A*. *flavus* to live through a stress environment. To determine if Dot1 participated in sclerotia formation, the Δ*dot1* mutant was cultured on WKM agar. The Δ*dot1* mutant was found to produce a larger amount of sclerotia (207 ± 1.73) than WT (150.67 ± 0.88) and the *dot1^C^* strain (152.67 ± 2.27) (*p* < 0.001) (Figure 4A,B). The qRT-PCR was performed to confirm the expressing levels of genes that were reported to be related with sclerotia formation, which indicated that *nsdC*, *nsdD* and *sclR* were all transcriptionally up-regulated in the Δ*dot1* mutant (Figure 4C), demonstrating that Dot1 negatively regulated sclerotia formation in *A*. *flavus*.

### 2.5. Dot1 Response to Multiple Stresses in A. flavus

**Figure 5:**
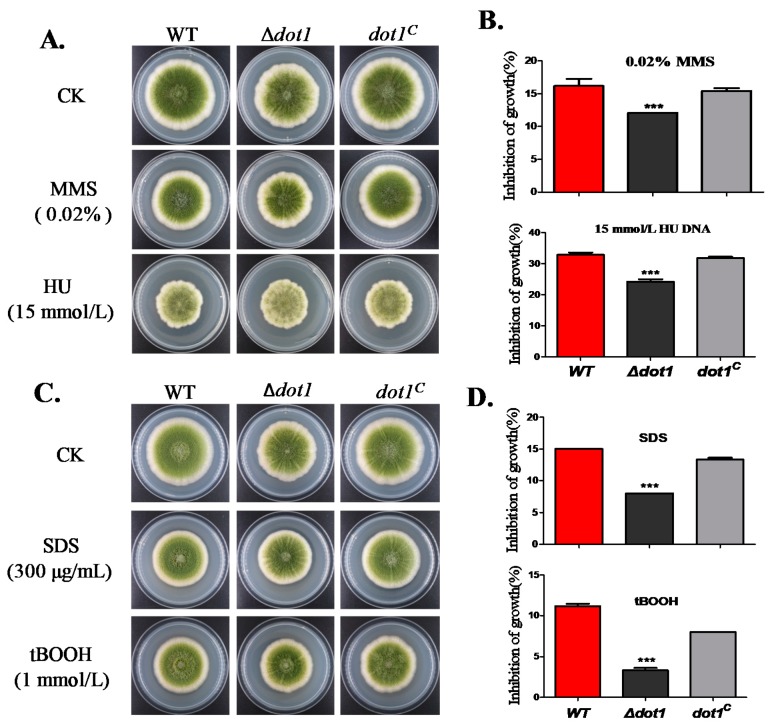
Growth of the WT, *dot1* and *dot1^C^* strains under multiple stresses. (**A**) Phenotype of WT, Δ*dot1* and *dot1^C^* strains when incubated within 0.02% methylmercuric sulfate (MMS) or 15 mM hydroxyurea (HU) for 3 d. (**B**) The growth inhibition rate of WT, Δ*dot1* and *dot1^C^* strains under genome integrity stress. The inhibition rate of growth was relative to the growth rate of each untreated strain, $\mathrm{Inhibition}\;\mathrm{of}\;\mathrm{growth}\;\mathrm{rate}=\frac{\left(\mathrm{the}\;\mathrm{diameter}\;\mathrm{of}\;\mathrm{untreated}\;\mathrm{strain}-\mathrm{the}\;\mathrm{diameter}\;\mathrm{of}\;\mathrm{treated}\;\mathrm{strain}\right)}{\mathrm{the}\;\mathrm{diameter}\;\mathrm{of}\;\mathrm{untreated}\;\mathrm{strain}}\times 100\%$; (**C**) Morphology of WT, Δ*dot1* and *dot1^C^* strains when incubated within 300 µg/mL SDS or 1 mmol/L tBooH; (**D**) The growth inhibition was quantified in (**C**). *** represent significantly different *p* ≤ 0.001. The experiments were performed with four biological replicates for each strain, and were repeated three times.

Histone post-translational modifications have been shown to contribute to DNA damage repair [28]. To determine the viability of *dot1* in response to genotoxicity stress, the strains were cultured on a PDA medium within 0.02% methylmercuric sulfate (MMS) or 15 mM hydroxyurea (HU) for 3 d. As shown in Figure 5A,B, the Δ*dot1* mutant showed less sensitivity to genotoxicity stress than the WT and *dot1^C^* strain. Since the Δ*dot1* mutant showed increased resistance to genotoxicity stress, we were also interested in addressing whether Dot1 responsed to other stresses. As we can see in Figure 5C,D, the WT and *dot1^C^* strains, but not Δ*dot1*, were much more sensitive to the cell-wall-damaging agent SDS (sodium dodecyl sulfate) and oxidative agent tBooH (tert-butyl hydroperoxide). All these results demonstrated that Dot1 has a potential role in response to multiple stresses in *A*. *flavus*.

### 2.6. Dot1 Contributes to Aflatoxin Biosynthesis

**Figure 6:**
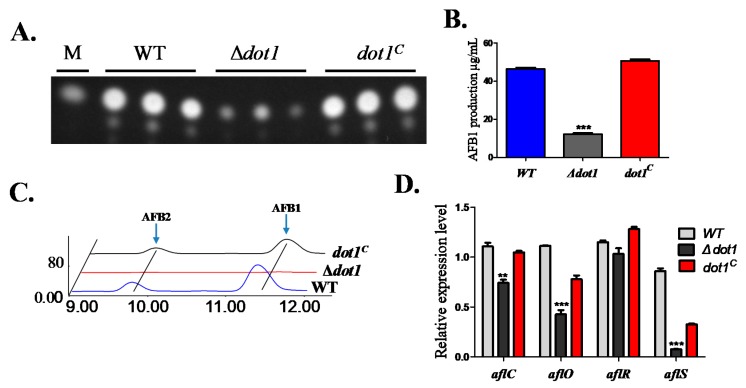
AF production of the WT, *dot1* and *dot1^C^* strains. (**A**) AF production was measured by Thin-Layer Chromatography (TLC) after being grown on PDA medium for 5 d at 29 °C in the dark. (**B**) Relative AF production in (**A**) was qualified. (**C**) HPLC analysis of aflatoxin produced by the WT, Δ*dot1* and *dot1^C^* strains after being grown on PDA medium for 5 d at 29 °C. (**D**) Transcriptional level of the AF-related genes *aflR*, *aflS*, *aflC(pksA)* and *aflO(omtB)* from WT, Δ*dot1* and *dot1^C^* strains. * and *** represent *p* ≤ 0.05 and *p* ≤ 0.001, respectively. The experiments were performed with three biological replicates for each strain, and were repeated three times.

A previous study showed that histone methylation is involved in secondary metabolite synthesis in many *Aspergillus* species [9]. Thus, we detected aflatoxin production—which is the most important secondary metabolite in *A*. *flavus*—in PDA agar media after being cultured for 5 d at 29 °C. The TLC results indicated that AF production was severely impaired in the Δ*dot1* mutant (Figure 6A,B). The result was further confirmed by HPLC analysis, which also showed a significant decrease in AFB1 production, as well as AFB_2_ production in the Δ*dot1* mutant (Figure 6C). The transcript levels of the AF-regulated genes(*aflR* and *aflS)* and the biosynthesis genes (*aflC* and *aflO*) in the Δ*dot1* mutant were determined by qRT-PCR, which demonstrated that *aflS*, *aflC* and *aflO*, but not *aflR,* were transcriptionally inhibited in the Δ*dot1* mutant (Figure 6D). All these results indicated that Dot1 might be involved in regulating aflatoxin biosynthesis in *A*. *flavus*.

### 2.7. Dot1 Contributes to t Crop Seeds Colonization

**Figure 7:**
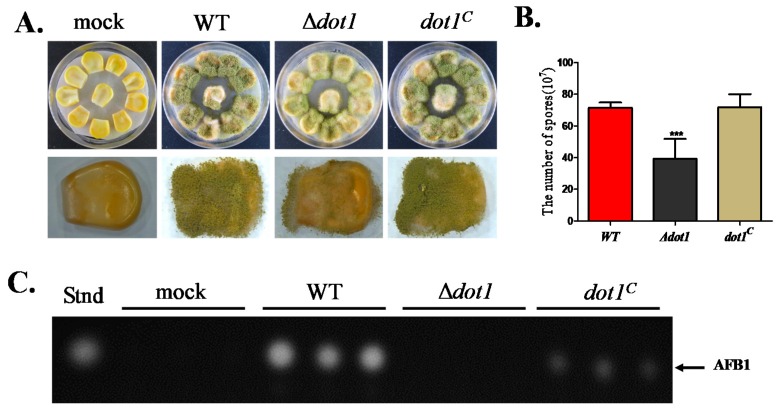
Seed infection of the WT, Δ*dot1* and *dot1^C^* strains. (**A**) Phenotype of fungal strains grown on living maize corn seeds; (**B**) The amount of conidia was determined from the infected seeds; (**C**) Mycotoxin production from infected maize corn seed was detected by TLC. *** represents *p* ≤ 0.001. The experiments were performed with five biological replicates for each strain, and were repeated three times.

*A. flavus* is notorious for its contamination of many important crops and plant seeds with aflatoxins. To assay the role of Dot1 in pathogenicity, maize corn seeds were inoculated with spore suspension from WT, Δ*dot1* and *dot1^C^* strains. The Δ*dot1* mutant showed a reduced ability to colonize maize seeds (Figure 7A), which also exhibited a significant drop in conidia production (Figure 7B). Mycotoxin production from the infected maize seeds was also detected, which showed that AF production was blocked in the Δ*dot1* mutant (Figure 7C). These data suggested that Dot1 was important for colonizing maize seeds.

### 2.8. Subcellular Localization of Dot1 in A. flavus

**Figure 8:**
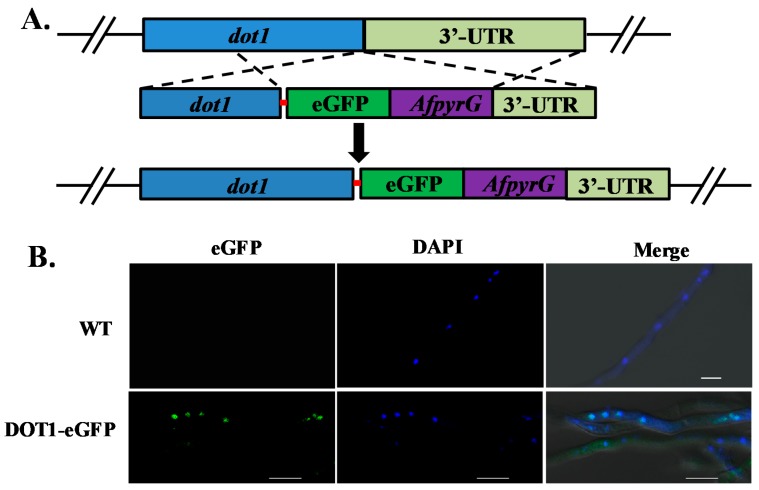
Location of Dot1-eGFP in *A*. *flavus*. (**A**) Construction strategy of *dot1(p)-dot1*-e*gfp* strain. A 5 Gly–Ala linker was shown as a red line. (**B**) Fluorescent image of Dot1-eGFP during the hyphae growth period, the nucleus was stained by 40, 6-diamidino-2-phenylindole (DAPI).

In order to determine the localization pattern of Dot1, we constructed an eGFP tag with a 5 Gly–Ala linker, and inserted the tag at the C-terminus of the Dot1 protein (Figure 8A). *AfpyrG* was used as the selective marker to screen the Dot1::eGFP transformants, which displayed no phenotype difference with the WT strain (data not shown), suggesting that Dot1-eGFP was fully functional. In the hyphae growth period, Dot1-eGFP was found to be mainly accumulated in the nucleus (Figure 8B), which was verified by 40, 6-diamidino-2-phenylindole (DAPI) stain, while no green fluorescence signal was found in the WT strain. All these results showed that Dot1 mainly functions in the nucleus.

## 3. Discussion

Histone methylation, as an important epigenetic modification, plays a vital role in the recruitment of other chromatin remodeling complexes and transcriptional machinery [16,29]. As an important HPTM, histone lysine methylation catalyzed by Dot1 is reported to be found from yeast to humans, which has been shown to be required for the DNA damage checkpoint [28,30,31]. However, studies of H3K79 methyltransferase in filamentous fungi are still rare, and no previous publications have yet reported the existence of these in *Aspergillus*. Here, we functionally characterized Dot1 in *A. flavus*, which shares a 33% overall identity with yeast Dot1. The locations and paralogs of *dot1* in *Aspergillus* genomes and the phylogenetic analysis of the Dot1 constructed in this study revealed that Dot1 was quite conserved in biological functions among *Aspergillus*.

Histone lysine methylation has been shown to provide an epigenetic layer for transcriptional regulation, and the methylation could lead to chromatin regions being active or repressive [12,13,14,15]. In this study, we found that disruption of the H3K79 methyltransferase gene *dot1* reduced *A*. *flavus* conidiation through down-regulating the transcriptional levels of the key transcription factors BrlA and AbaA [32]. Conidiation and sclerotial formation have been shown to stay balanced in *Aspergillus* [1]. Here, we also found that inactivation of *dot1* up-regulated the expression levels of the sclerotia-related genes *nsdC*, *nsdD* and *sclR*, as a result, increasing sclerotia reproduction. Sclerotia is considered to be a survival structure for adapting to stress environments, here we also found that the Δ*dot1* mutant showed less sensitivity to genotoxicity stress, cell wall-damaging agents and oxidative stress. All this suggested that methyltransferase Dot1 is involved in fungal development and stress response.

In *A. flavus*, an AF gene cluster has been shown to be located near the telomeres [19,20], which are associated with specific chromatin structures [19,21]. Our former studies had shown that DNA methyltransferase and histone acetylation contribute to AF biosynthesis in *A*. *flavus* [10,33], but not for arginine methyltransferase [25], which indicates that HPTMs are involved in the regulation of AF biosynthesis. HPTMs such as acetylation, methylation, and ubiquitination have been linked to transcriptional silencing in the telomere region. The inactivation of H3K79 methylation or H3K4 methylation is quite important for heterochromatin formation and the maintenance of the silencing regions of chromatin in budding yeast [21,22,23]. An abnormal expression of the H3K79 methyltransferase gene *dot1* in yeast and mammalian cells leads to a decrease in gene silence [23,24]. Here, we found that the most important secondary metaboliteaflatoxin in *A*. *flavus* was severely impaired in the *dot1* deficient mutant compared to the WT strain. In *A*. *nidulans*, studies have shown that H3K9 methylation and heterochromatin protein-binding are involved in the activation of the sterigmatocystin gene cluster [34]. Simultaneously, we found that the transcription of genes relevant to aflatoxin biosynthesis, including the structural genes *aflC* and *aflO* and the regulator gene *aflS,* were prominently suppressed in the Δ*dot1* mutant. However, the expression levels of the AF global regulator gene *aflR* showed no difference between the Δ*dot1* mutant and WT strain. Dot1 might affect AF biosynthesis by regulation of the activation of AflS, which could affect the activity of AflR in *A*. *flavus*. Therefore, it could be suggested that Dot1 is involved in activating the aflatoxin biosynthesis-relevant gene cluster.

In many plant pathogens, including *Magnaporthe oryzae* and *Fusarium graminearum*, the histone lysine methylation is known to function in fungal virulence [35,36]. Many seed crops, such as maize corn and peanuts, can be potentially colonized by *A*. *flavus*, which could sporulate on injured seeds and subsequently contaminate the hosts with aflatoxin. Here the *dot1^C^* complemented strain, although normal in its phenotype, had a defect in the activation of *aflS* and AF production during colonization, indicating that Dot1 in the complemented strain might not fully function. However, we found that the Δ*dot1* mutant shows a significant reduction in pathogenicity in *A*. *flavus*. Although the physiological significance of these H3K79 methylation events remains unknown, the reduction of conidiation and aflatoxin biosynthesis as a result of the inactivation of Dot1 might be related to fungal virulence in *A. flavus*.

## 4. Conclusions

We identified a novel and functional methyltransferase Dot1 in *A*. *flavus*, and we found that Dot1 contributes to conidiation, AF metabolism and fungal virulence in *A. flavus*. Our preliminary results suggest valuable information that could advance our understanding of H3K79 methylation modification in the regulation of AF biosynthesis and fungal pathogenicity in *A. flavus*, and would provide a potential target for new control strategies of this fungal pathogen.

## 5. Materials and Methods

### 5.1. Strain and Culture Conditions

The *A. flavus* strains used in this study are listed in Table 1. Potato dextrose agar (PDA, BD Difco, Franklin, NJ, USA) was used for the growth and conidiation assays, supplemented with the appropriate amounts of uridine (5 mM), uracil (5 mM), or pyrithiamine (100 ng/mL) when necessary. The modified Wickerham medium (WKM) was used for the sclerotial production analysis [37]. After being grown for 7 days, the cultures in the WKM plates were washed with 70% ethanol to visualize sclerotia. PDA agar supplemented with 100 μg/mL sodium dodecyl sulfate (SDS), 2.0 mM/L tert-butyl hydroperoxide (tBooH), 15 mM hydroxyurea (HU), or 0.02% methyl methanecsulfonate (MMS) were used to determine sensitivities to multiple stresses. All the experiments were performed with technical triplicates, and were repeated three times.

### 5.2. Generation of Gene Deletion and Complementation Strains

The *dot1* deficient mutant (Δ*dot1*) and the Δ*dot1* complemented strain (*dot1^C^*) were generated by using a previously described method [33]. The primers used for gene knockout are listed in Table 2. A 1189-bp 5′ flanking fragment of *dot1* was amplified with primers *dot1*/P1 and *dot1*/P3, and a 1033-bp 3′ flanking fragment of *dot1* was amplified using primers *dot1*/P4 and *dot1*/P6. A *pyrG* selective marker was amplified from *A. fumigatus* genomic DNA with primers *pyrG*/F and *pyrG* /R. The nested primers *dot1*/P2 and *dot1*/P5 were used to generate the *dot1* deletion construct containing the 5′ and 3′ flanking region and the *pyrG* selection marker. The fusion PCR products were purified and transformed into the protoplasts of *A. flavus* CA14 PTs strain using an established procedure (Yang et al., 2016). Transformants were screened by PCR with primer *dot1*-ORF/F and *dot1*-ORF/R (Table 2), and further confirmed by Southern blot analysis. 

To complete Δ*dot1*, a 2.9-kb PCR product (1.6-kb *dot1* coding sequence, 0.8-kb upstream sequence and 0.5-kb *dot1* terminator region) was amplified from *A*. *flavus* wild-type genomic DNA using primers *dot1C*M/F and *dot1C*M/R. The purified PCR product and the linearization pPTRI (Takara, Tokyo, Japan) vector were then recombined with T4 DNA ligase. The recombinant pPTR-*dot1* was transformed into Δ*dot1* protoplasts. The pyrithiamine-resistant transformants were screened by PCR, and confirmed by reverse transcription PCR (RT-PCR).

### 5.3. Aflatoxin Analysis

The WT, Δdot1 and dot1C strains were incubated into 20 mL potato dextrose broth (PDB) medium in the dark at 29 °C for 5 days. Then the cultures were used for AF extraction. AF extraction was performed as previously described in [33]. Thin layer chromatography (TLC) and high performance liquid chromatography (HPLC) were both used to measure AF production with the method described in [33]. 

### 5.4. Maize Corn Infection Assay 

The maize seed colonization assay was performed as described previously in [6,39]. The maize seeds were co-cultured with conidia of the WT, Δ*dot1* and *dot1^C^* strains 29 °C for 5 d. After five days incubation, the infected seeds were collected in 50 mL Falcon tubes, then mixed with 15 mL of sterile 0.05% Tween 80, followed by 2 min vortex to release the spores. A 100 μL aliquot of spores was removed, diluted, and counted haemocytometrically. 15 mL of chloroform were added to the Falcon tubes, and the tubes were shaken at 29 °C for 30 min. 10 mL of the organic layer was removed, dried down, and resuspended in 1 mL chloroform. Finally, AF production was detected by TLC.

### 5.5. Gene Expression Detection

The gene expression level was detected by qRT-PCR. 48 h old mycelia of *A*. *flavus* were collected, washed, and lyophilized from PDA medium. Total RNA was isolated according to the protocol previously described by Yang et al. [6]. SYBR Green Supermix (Takara) was used for the qRT-PCR reaction with the PikoReal 96 Real-time PCR system. The 2^−ΔΔCT^ method was used to calcite the relative quantification of each transcript [40]. The qRT-PCR primers are listed in Table 3.

### 5.6. Generation of Dot1-eGFP Strain

The former approach for protein location was performed to generate the Dot1-GFP strain [6,41]. To generate the dot1-eGFP construct, a 1068 bp *dot1* ORF without the termination codon (TAG) and a 1033-bp 3′ flanking fragment of *dot1* was amplified from *A. flavus* WT strain gDNA using the primer pairs *dot1*-eGFP, P1-*dot1*-eGFP/P3 and *dot1*/P4, *dot1*/P6, respectively. The primers *dot1*-eGFP/P2 and *dot1*-eGFP/P5 were used to generate the Dot1-eGFP fusion PCR cassettes containing the upstream and downstream region, *egfp* and the *pyrG* selection marker. The purified fusion PCR products were inserted into the protoplasts of the *A. flavus* CA14 PTs strain, and the selected transformants were then confirmed by PCR.

### 5.7. Microscopic Determination of Dot1-eGFP Location

To assay the subcellular location pattern of Dot1-eGFP, 12 h growth mycelial was collected and analyzed using a Leica SP8 confocal laser scanning microscope. Dual-channel imaging was used to sequentially image cells labeled with DAPI (excitation: 405 nm, emission bandwidth: 420–460 nm), and eGFP (excitation: 488 nm, emission bandwidth: 525/565 nm). 

### 5.8. Statistical Analysis

Statistical analysis was performed using ANOVA and least significant difference (LSD) tests to determine significant differences among group means. A *p*-values less than 0.05 was regarded as statistically significant.

## Figures and Tables

**Table 1:** *A*. *flavus* strains used in this study.

Strain	Genotype Description	Reference
*A. flavus CA14 PTs*	Δ*ku70*, Δ*pyrG*	[38]
wild-type	Δ*ku70*, Δ*pyrG*::*AfpyrG*	This study
*Δdot1*	Δ*ku70*, Δ*dot1*::*AfpyrG*	This study
*Δdot1^C^*	Δ*ku70*, *Δdot1*:: *AfpyrG*, *dot1*(*p*)::*dot1*::*ptrA*	This study
G*dot1*	Δ*ku70*, *dot1(p)*::*dot1-egfp*::*AfpyrG*	This study

**Table 2:** Primers used for gene deletion and complementation.

Primers	Sequence (5′-3′)	Application
*dot1*/P1	CATTGAGATGGACGAGGACG	*dot1* deletion and probe
*dot1*/P3	GGGTGAAGAGCATTGTTTGAGGCGTTGACCGAGCGGAAATGT	
*dot1*/P4	GCATCAGTGCCTCCTCTCAGACCACTACGCGGTTACCGAGAC	
*dot1*/P6	CGACAGAAAGCCATAATGAAAT	
*dot1*/P2	CGAGGACGACAATGACTACAC	
*dot1*/P5	GAGTTGAAGGGAAAGGCTAAA	
PyrG/F	GCCTCAAACAATGCTCTTCACCC	*pyrG selective marker*
PyrG/R	GTCTGAGAGGAGGCACTGATGC	
P801/R	CAGGAGTTCTCGGGTTGTCG	
*dot1*-ORF/F	TTCTTACAGTATCCGAGTGC	*dot1* mutant screen
*dot1*-ORF/R	TTCATGTCCAGGAAGTGGTT	
CM-*dot1*/F	CTATGACCATGATTACGCCAACTATGACCATGATTACGCCAAGCTTAGCCTAAGCAGCAGGTGAAGC	*dot1* complementation construct
CM-*dot1*/R	CCAGTGAATTCGAGCTCGGTACCGATGGCAAGGATGGGCAAAG	
eGFP-pyrG/F	GGAGCTGGTGCAGGCGCTGGAGCCGGTGCCATGGTGAGCAAGGGCGAGGA	*egfp*
eGFP-pyrG/R	GGGTGAAGAGCATTGTTTGAGGCTTACTTGTACAGCTCGTCCATG	
*dot1*-eGFP/P1	ATTCTTACAGTATCCGAGTGC	*dot1-gfp* tag construct
*dot1*-eGFP/P3	GGCTCCAGCGCCTGCACCAGCTCCACCCATGCTCTCTGCAAAAG	
*dot1*-eGFP/P2	AAAATTACATACCGGAGGACG	

**Table 3:** Primers used for RT-PCR.

Primers	Sequence (5′-3′)	Application
*brlA/QF*	GCCTCCAGCGTCAACCTTC	*brlA* qRT-PCR
*brlA/QR*	TCTCTTCAAATGCTCTTGCCTC	
*abaA/QF*	CACGGAAATCGCCAAAGAC	*abaA* qRT-PCR
*abaA/QR*	TGCCGGAATTGCCAAAG	
*nsdC*/QF	GCCAGACTTGCCAATCAC	*nsdC* qRT-PCR
*nsdC*/QR	CATCCACCTTGCCCTTTA	
*nsdD*/QF	GGACTTGCGGGTCGTGCTA	*nsdD* qRT-PCR
*nsdD*/QR	AGAACGCTGGGTCTGGTGC	
*sclR*/QF	CAATGAGCCTATGGGAGTGG	*sclR* qRT-PCR
*sclR*/QR	ATCTTCGCCCGAGTGGTT	
*aflC*/QF	GTGGTGGTTGCCAATGCG	*aflC* qRT-PCR
*aflC*/QR	CTGAAACAGTAGGACGGGAGC	
*aflR*/QF	AAAGCACCCTGTCTTCCCTAAC	*aflR* qRT-PCR
*aflR*/QR	GAAGAGGTGGGTCAGTGTTTGTAG	
*aflS*/QF	CGAGTCGCTCAGGCGCTCAA	*aflS* qRT-PCR
*aflS*/QR	GCTCAGACTGACCGCCGCTC	
*aflO*/QF	GATTGGGATGTGGTCATGCGATT	*aflO* qRT-PCR
*aflO*/QR	GCCTGGGTCCGAAGAATGC	
Actin/QF	ACGGTGTCGTCACAAACTGG	*actin* qRT-PCR
Actin/QR	CGGTTGGACTTAGGGTTGATAG	
